# Immune inflammation markers and physical fitness during a congested match play period in elite male soccer players

**DOI:** 10.1038/s41598-024-81225-0

**Published:** 2024-12-05

**Authors:** Karim Saidi, Abderraouf Ben Abderrahman, Ismail Laher, Anthony C. Hackney, Rawad El Hage, Ayoub Saeidi, Benoit Bideau, Urs Granacher, Hassane Zouhal

**Affiliations:** 1 UFR Sciences and Techniques of Physical and Sports Activities, Toulon University, Toulon, France; 2 Higher Institute of Sport and Physical Education, Ksar-Said, University of Manouba, Manouba, Tunisia; 3 Tunisian Research Laboratory “Sport Performance Optimization”, National Center of Medicine and Science in Sports, Tunis, Tunisia; 4 Department of Anesthesiology, Pharmacology and Therapeutics, The University of British Columbia, Vancouver, Canada; 5 Department of Exercise & Sport Science, University of North Carolina, Chapel Hill, NC USA; 6 Department of Physical Education, Faculty of Arts and Sciences, University of Balamand, PO Box 100, Tripoli, Lebanon; 7 Department of Physical Education and Sport Sciences, Faculty of Humanities and Social Sciences, University of Kurdistan, Sanandaj, Kurdistan Iran; 8 Movement, Sport, Health and Sciences laboratory (M2S), University of Rennes 2, Rennes, France; 9 Department of Sport and Sport Science, Exercise and Human Movement Science, University of Freiburg, Freiburg, Germany; 10 Institut International des Sciences du Sport (2I2S), Irodouer, 35850 France

**Keywords:** Congested calendar, Integrative immune markers, Overtraining, Soccer, White blood cells, Biochemistry, Immunology, Physiology

## Abstract

**Background/Objective:**

Cellular immune markers of inflammation such as neutrophil-to-lymphocyte ratio (NLR), platelet-to-lymphocyte ratio (PLR) and systemic immune inflammation index (SII) are frequently used in patient care. The adoption of these markers to elite sports, e.g. soccer could be beneficial when monitoring training and aiming to maximize physical fitness. This study investigated cellular immune inflammation markers and physical fitness in elite male soccer players in relation to changes in training and match exposure during a congested match play period.

**Methods:**

Fifteen elite male soccer players were evaluated three times (T1, T2, and T3) over 12 weeks (T1–T2: six weeks uncongested period of match play and T2–T3: six weeks congested period of match play). Players performed vertical jump tests (squat jumps [SJ], countermovement jumps [CMJ]), the 20-meter sprint test, and the Yo-Yo intermittent recovery test (YYIRL1) at T1, T2 and T3. Measurements included counts of leucocytes and its subtypes, as well as platelets. Cellular immune inflammation markers (NLR, PLR and SII) were calculatedat T1, T2, and T3. Training session rating of perceived exertion was also recorded on a daily basis.

**Results:**

Significant increases in leucocyte, neutrophil, eosinophil, basophil and monocyte counts occurred at T3 compared with T2 (0.002 < *p* < 0.04, -0.56 < ES < -0.40) and T1 (-0.78 < ES < -0.49). Lymphocyte counts were lower at T3 as compared to T2 and T1 (*p* = 0.038, -0.48 < ES <-0.25), while NLR, PLR and SII were greater at T3 compared to T2 (0.001 < *p* < 0.015, -1.01 < ES < -0.44) and T1 (-0.99 < ES < -0.21). There was a negative correlation between YYIRL1 performance with NLR (*r*= -0.56; *p* = 0.02), PLR (*r*=-0,44, *p* = 0.015), and SII (*r*= -0.63; *p* = 0.01) after the congested period of match play (i.e., T3). Values for maximal oxygen uptake (VO_2max_), estimated from the YYIRL1 test, negatively correlated with NLR (*r*= -0.56; *p* = 0.02), PLR (*r*=-0,44, *p* = 0.015), and SII (*p* = 0.01; *r*= -0.63). There was a positive correlation between NLR, and SII with workload parameters. In addition, a clear positive correlation was observed between NLR and SII with competitive loadinstead (r= [0.59–0.64; p˂ 0.001), training load (TL) (r= [0.65–0.68]; p˂ 0.001), session rating of perceived exertion (S-RPE) (r= [0.65–0.68]; *p* = 0.001), and training volume (r= [0.60–0.61; *p* = 0.001).

**Conclusion:**

An intensive period of congested match play significantly alterated immune cell counts and cellular markers of inflammation (NLR, PLR and SII). Changes in NLR and SII were related to workload parameters, suggesting the usefulness of these markers in regulating training intensity and competitive load. An association between physical fitness (YYIRL1, VO_2max_) and NLR, PLR and SII suggests that these biomarkers are promising tools to monitor aerobic physical fitness of elite soccer players during congested periods of match play.

## Introduction

Professional soccer has evolved in terms of game intensity (e.g., increase in running distance, number of runs and sprints, high-speed actions) and the number of matches played during the season. For instance, elite players can play up to 75 matches per season^1,2^. This can result in elite players being exposed to periods of match congestion involving up to eight matches per month, leaving little time for adequate recovery between games^3,4^, which can strain physiological and immune systems that can impact performance^5,6^ and also increase the risk of overtraining and injury^7^ as well as infection^8^. In addition, the congested match calendar poses difficulties not only for the players but also for the technical and medical staff, thus requiring increased monitoring of training and competition loads^9^, to optimize training as well as manage or adjust upcoming training and competition loads^10^.

The close monitoring of players through the usage of circulating biomarkers can prevent overtraining, and at the same time improve physical fitness during congested match schedules^11^. When searching for markers of exercise-induced inflammation, immunological parameters may be useful as the immune system participates in an inflammatory response^12^. Frequently used immune inflammation markers in exercise physiology are leucocyte counts and the percentages of subpopulations, granulocytes (neutrophils, eosinophils, and basophils), monocytes, and lymphocytes^13^. An elevated leucocyte count is often indicative of an existing infection or inflammation, while a shift between different leucocyte subsets could be due to physical training^14^, or be a symptom of insufficient post-exercise recovery^15^. In soccer, leucocyte counts are mainly used to identify acute fatigue and post-exercise recovery^16^.

An additional array of cellular immune inflammation markers of disease-related inflammation with integrative aspects have recently been proposed, including the neutrophil-to-lymphocyte ratio (NLR), platelet-to-lymphocyte ratio (PLR), and the systemic immune inflammation index (SII)^11^. By integrating the kinetics of the two largest leucocyte subsets into one single parameter, NLR could have great potential as an inflammation marker with increased values indicating ongoing inflammatory processes. In contrast to NLR, PLR is not only based on leucocyte subsets but also takes platelet counts into consideration. Besides the well-known role of platelets in hemostasis and thrombosis, they also exhibit various pro-inflammatory properties, underscoring their value as markers of inflammation^17^. Additionally, the SII is a cellular immune inflammation marker that integrates the kinetics of NLR and PLR into a single parameter. While NLR and PLR are calculated as ratios of two different blood cell populations, the SII considers three populations by multiplying the NLR with platelet counts. These integrative markers are mainly used in oncology^18^, neurology^19^, or cardiovascular disease^20^, but less frequently in physically active individuals or athletes^21^.

The use of NLR, PLR and SII is still limited in exercise physiology, but is attracting more attention because of growing evidence indicating a moderate-to-strong correlation of NLR, PLR, and SII with other well-established inflammatory markers such as leucocytes^22^, C-reactive-protein^23^, and interleukin-6^24^. In addition, the integrative value of the markers is that they can be used as measures to elucidate patient’s or individual’s inflammatory status^25^. Exercise-induced inflammation likely could be assessed in a similar manner, and be a tool used for customizing training programs to individual recovery needs.

Likewise, seeking interactions between cellular immune inflammation markers and physical fitness and identifying changes in these biomarkers during a competitive soccer season may be helpful to identify periods of increased infection risk or overtraining. In addition, these markers can also provide insights on managing training and match participation during a congested match play period^11,26^. In this study, we aimed to examine changes in cellular immune inflammation markers, and physical fitness across different periods of the soccer season and whether these changes are related to the training load and frequency of matches played during a competitive soccer season. We hypothesized that the cellular immune inflammation markers may be altered after the congested periods of match play, which in turn, could cause negative changes in players’ physical fitness which would be evident through the associations between selected measures^11,26^.

## Methods

### Participants

Twenty four elite soccer players from the same soccer team competing in the Tunisian Premier League voluntarily agreed to participate in this study. Inclusion criteria used to recruit study participants were: a) all players were healthy (not suffering from fever, asthma, allergies) and had not suffered any injuries, infections, and/or illness in the preceding four weeks, (b) the players had to participate in at least 85% of the scheduled training sessions and matches during the study period, and (c) commit to completing all study assessments. A total of 15 players were eligible for inclusion in this study; these players had the greatest match exposures over the experimental period, and engaged in supervised soccer training and in national competitions for at least five years (Table 1). The participants received written and verbal explanations of the study, informing them of all risks and benefits associated with study participation, and written informed consent was obtained. The local ethics committee of the Scientific Council of the University of Rennes 2, France and the medical unit of the soccer club (Jeunesse sportive Kairouannaise, JSK) provided approval (ethical approval code: 02/2019) for all experimental procedures and the study procedures were in agreement with the Declaration of Helsinki.

**Table 1:** Characteristics of the players.

Parameters	Mean [SD]
Age (years)	20.5 [0.8]
Height (m)	177 [4]
Body mass (kg)	72 [5.2]
BMI (kg/m^2^)	22.7 [1.1]
BF (%)	8.5 [2.4]
Training adherence (%)	89.2 [2.6]
Total competitive minutes (min)	1649.3 [293.4]

Data are mean and SD. **BMI**, Body mass index; **BF**, Body fat.

### Study design

**Fig. 1:**
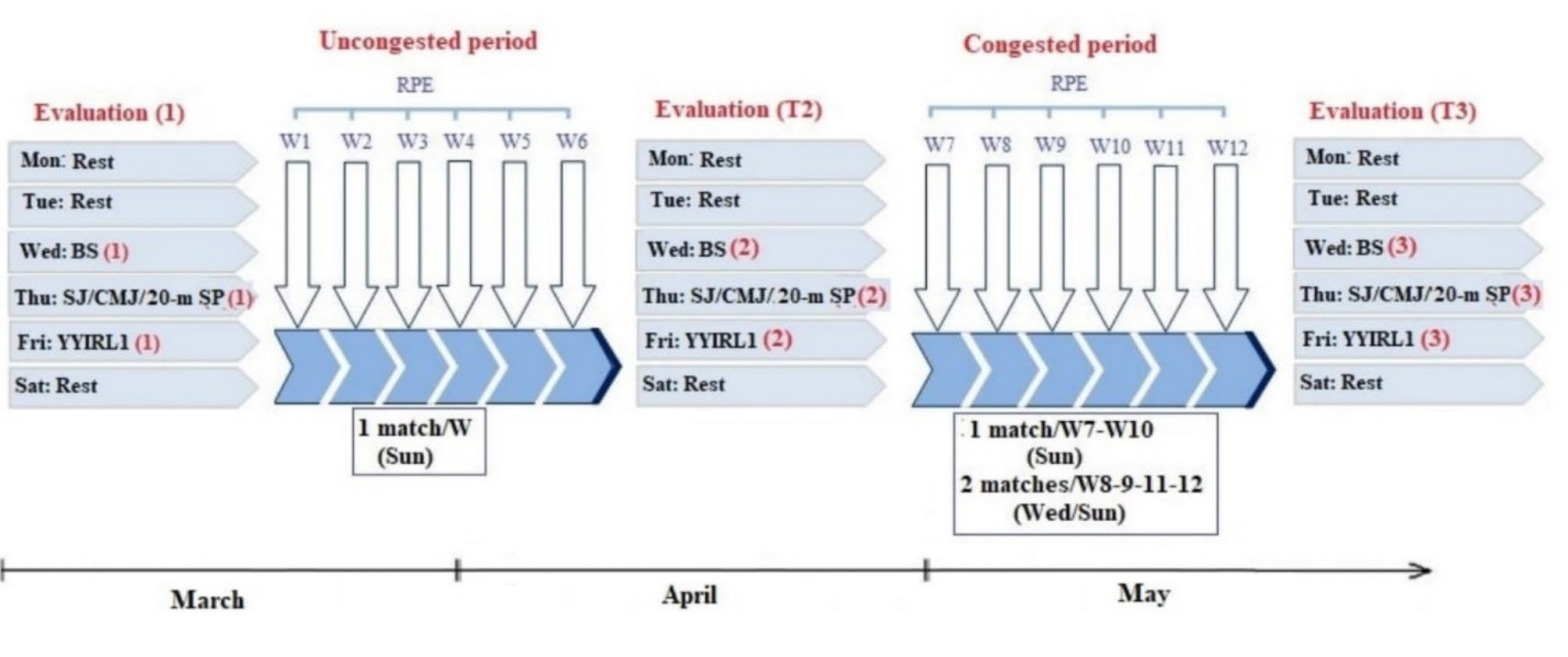
Experimental design. **Abbreviations**: **W**, week; **BS**, blood sample;** CMJ**, countermovement jump; **YYIRL1**, Yo-Yo intermittent recovery test-level 1; **Sun**, Sunday; **Mon**, Monday; **Tue**, Tuesday; **Wed**, Wednesday; **Thu**, Thursday; **Fri**, Friday; **Sat**, Saturday; **Sun**, Sunday;** RPE**, rating of perceived exertion;** SP**, sprint; **SJ**, squat jump.

A schematic overview of the study design is shown in Fig. 1.The study started in March and lasted till May 2021. The participating players were monitored for 12 weeks, starting six weeks after the beginning of the second competitive period following the winter break. Players trained five times/week and played in one match during this period. All players were evaluated three times during this study: T1-in the middle of the uncongested period (week 1); T2- the end of the uncongested period (before the congested period; week 7); T3**-**after the congested period (week 13).

Blood samples to measure resting leucocyte counts were collected on the first day. Participants performed three physical fitness tests in random order on the second day: the squat (SJ)^29^ and countermovement jump (CMJ)^29^, the 20-meter linear sprint test^30^. Rest periods between tests lasted 5 min to allow players sufficient recovery time. Participants performed the Yo-Yo intermittent recovery test level 1 (YYIRL1)^36^ on the third test day. All physical fitness tests were performed in the afternoon, three hours after taking a standard light meal. The players were familiar with the physical fitness tests used as these tests are routinely applied during the soccer season. Players were asked to follow the same nutritional plan 24 h before each test session to minimize diet-induced performance changes. Ratings of perceived exertion (OMNI scale 1–10) were recorded on a daily basis to quantify training load (session ratings of perceived exertion × training session time in minutes, and competitive load (session ratings of perceived exertion × match time).

### Training program

The training program used during the uncongested and congested periods of match play is summarized in Table 2. The training program was set by the team coaches and was not influenced by the study protocol.

**Table 2:** Training programs during uncongested and congested weeks of match play.

	Uncongested period	Congested period
Day	Weekly program during the uncongested period and during the period of congested match play (when playing one match per week). Weeks 1 to 6 and weeks 7–10	Weekly program during the congested period of match play (when playing two matches per week). Weeks 8-9-11-12
Monday	Full recovery	Warm up, Low-to-moderate intensity aerobic exercise Technical training
Tuesday	Warm up, Technical training, Low-to-moderate intensity aerobic exercise Small-sided games	Full recovery
Wednesday	Warm up, Strength training, Tactical training, Small-sided games	Match
Thursday	Warm up, Tactical training, Speed training over short distances Small-sidedgames	Warm up, Tactical training, Speed training over short distances Small-sided games
Friday	Warm up, Technical training, Speed training over long distances Soccer specific training	Warm up, Technical training, Speed training over long distances Soccer specific training
Saturday	Warm up /technical training, Speed training over short distances Soccer specific training	Warm up /technical training, Speed training over short distances, Soccer specific training
Sunday	Match	Match

### Blood sampling

Blood samples were collected between 8:00 and 9:00 am on the test days and always 72 h after the previous playing game. A rest-recovery period of 12 h was scheduled the day before the samples were taken and blood samples were collected after an overnight fast. Analysis of the blood samples was performed by authorized laboratory technicians at the medical analysis laboratory. Blood (30 ml) was collected in seated position via the antecubital vein into 10 ml vacutainer tubes (Vacuette R, Greiner bioOne, France) with ethylenediaminetera-acteicacid (EDTA) and used to determine leucocyte, neutrophil, lymphocyte, monocyte, eosinophil, basophil, and platelet counts using the flow cytometry technique (CelltacES, Japan). Cellular immune inflammation markers were calculated according to the following equations:^26,27^

$$NLR\left( {A.U} \right) = Neutrophilcounts\left( {m{m^3}} \right)\:/Lymphocytes\:counts\:\left( {m{m^3}} \right).$$

$$PLR\:\left( {A.U} \right) = Platelet\:counts\:(/m{m^3})/Lymphocyte\:counts\:\left( {m{m^3}} \right).$$

$$SII\:\left( { \times \:{{10}^3}/m{m^3}} \right) = NLR\:\left( {m{m^3}} \right) \times \:Platelet\:counts\left( {m{m^3}} \right).$$

### Physical fitness tests

#### Yo-Yo intermittent recovery test level 1

The YYIRT1 was used to assess players’ ability to repeat high-intensity exercises^36^. Players ran two 20-m linear sprints after a start signal, separated by a 10 s recovery period controlled by an audio metronome from a calibrated CD player. The time between start signals decreased over the stages and the test was ended if the players failed to reach the finish line in time on two occasions, or the player decided that he could no longer run at the required pace. The total distance covered (in meters) during the test was recorded.

In addition, the maximal oxygen uptake (VO_2max_) was estimated with the following equation:$V{O_{2\max }}(ml.K{g^{ - 1}}.{\min ^{ - 1}}) = \:YYIRL1\:dis\tan ce\:\left( m \right) \times \:0.0084 + 36.4.$^36^.

#### Vertical jump tests

Each player performed two different maximal jumps in random order: athletes started the SJ from a static semi-squatting position with knee angles at approximately ∼90°. Players started the CMJ from a standing position and subsequently performed a vertical jump at maximal effort using a slow stretch-shortening cycle (SSC) with a ∼90° knee flexion. Players were encouraged to jump as high as possible during both tests. Vertical jump height was evaluated using an optoelectric system (Opto-Jump Microgate – Italy). Jump height was calculated according to the following equation: $\:Jump\:height = 1/8 \times \:g \times \:{t^2}$, where g is the acceleration due to gravity and t is the flight time^29^.

#### 20-meter linear sprint test

The 20-m linear sprint test was conducted to assess players’ speed capacity. Validity and reliability of the linear sprint tests have been confirmed for adult soccer players in a recent systematic review^30^. Players were asked to start with feet split after a 15-minute warm-up, using the preferred leg in front. Players were asked to perform their best efforts possible and to run at full speed until the finish line. The 20-m linear sprint test was conducted using an infrared photoelectric cell (Cell Kit Speed Brower) with an accuracy of 0.01 s. Players performed the 20-m sprint test three times with at least 3 min rest between each trial, and their performance was recorded in seconds for further analysis.

### Monitoring training and competitive loads

Subjective measures were used to estimate internal loads, and a CR-10 scale was applied to quantify the rate of perceived exertion (RPE) for each player^31^. Based on the CR-10 scale, a value of 1 refers to“very light activity” and a value of 10 refers to “maximal exertion”. Training and competitive load monitoring were performed 30 min after each training session and match. All players were familiarized with the CR-10 scale and the RPE scores were multiplied by the duration (in minutes) of the session or match to calculate internal measures of training and competitive load. Total load or global load were calculated as the sum of training load + competitive load. Data were categorized into two competitive periods of training and games, i.e., the uncongested and the congested periods of match play.

### Statistical analyses

Statistical analyses were conducted using statistical software SigmaStat (version 3.5; Systat, Inc). All parameters were tested and confirmed for data normality using the Kolmogorov–Smirnov test before further statistical analyses were conducted. Paired-t-tests for dependent samples were used to identify differences in workload parameters (GL: Global load; TL: Training load; CL: Competitive load, Training volume (TR-V), session-rating of perceived exertion (S-RPE) between the congested (between T2-T3) and the uncongested periods (between T1-T2) of match play. Mixed linear models with repeated measures were used to determine differences in immune inflammation markers and physical fitness parameters measured at different time points (T1, T2, T3). Bonferroni-corrected post-hoc tests were computed. For each parameter, coefficients of variation (CV; CV = ([SD/mean] ×100) were calculated for the whole group and also intra-individually during the competitive periods.

A simple linear regression was used to analyze the relationships between the different parameters. Coefficients of determination (R^2^) were used to interpret the meaningfulness of the relationships and within subject correlations were calculated between cellular immune inflammation markers with workload parameters and physical fitness during the competitive periods. The magnitude of correlation coefficients was considered as trivial (*r* < 0.1), small (0.1 < *r* < 0.3), moderate (0.3 < *r* < 0.5), large (0.5 < *r* < 0.7), very large (0.7 < *r* < 0.9), nearly perfect (0.9 < *r* < 1.0), and perfect (*r* = 1.0). When 95% confidence interval (CI) overlapped positive and negative values, the effect was deemed to be unclear. The 95% CIs and effects sizes (ESs) were calculated to compare differences in mean values for all analyzed parameters. When calculating ES, pooled SDs were used since no control group was available (Cohen’s d = [M1–M2]/ SD_pooled_). ES with values of 0.2, 0.5, and 0.8 were considered to represent small, medium, and large differences, respectively^33^. Statistical significance was set at *p* < 0.05 for all analyses^34^.

## Results

Issues related to player injuries and/or player absences resulted in 37.5% (*n* = 9) of missing cases for all parameters. Valid cases represent 62.5% which equals *n* = 15.

The players performed 30 sessions with an overall training volume of 45 hours and played six matches with an average playing time of 350 ± 53.5 minutes during the six-weeks uncongested period. The soccer players performed 24 sessions with a training volume of 36 hours and played ten matches with an average playing time of 629.6 ± 133.9 during the congested period.

**Fig. 2:**
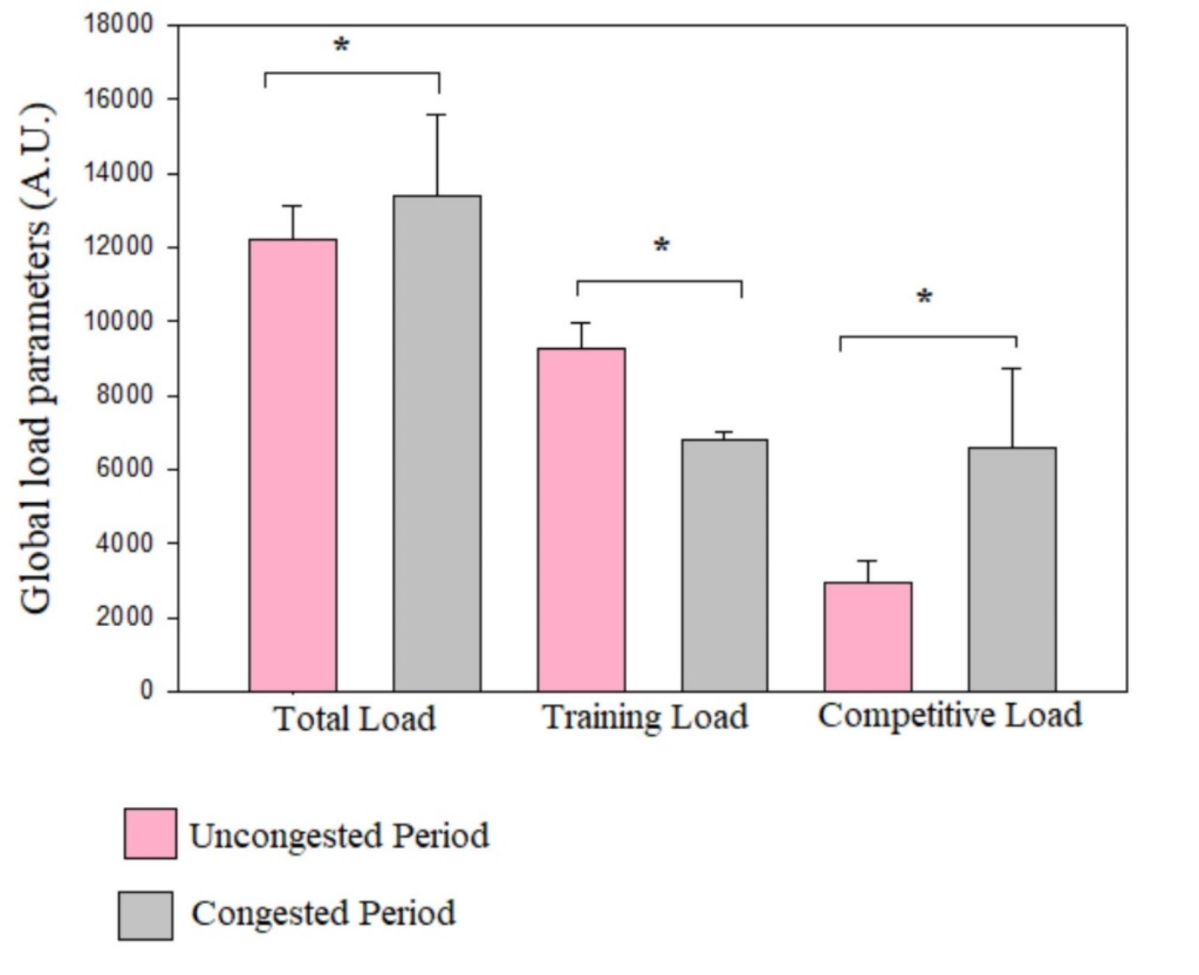
Team measures of global load parameters during the uncongested and congested periods of match play. **Abbreviations: AU**, arbitrary units. *significant difference between uncongested and congested periods.

**Fig. 3:**
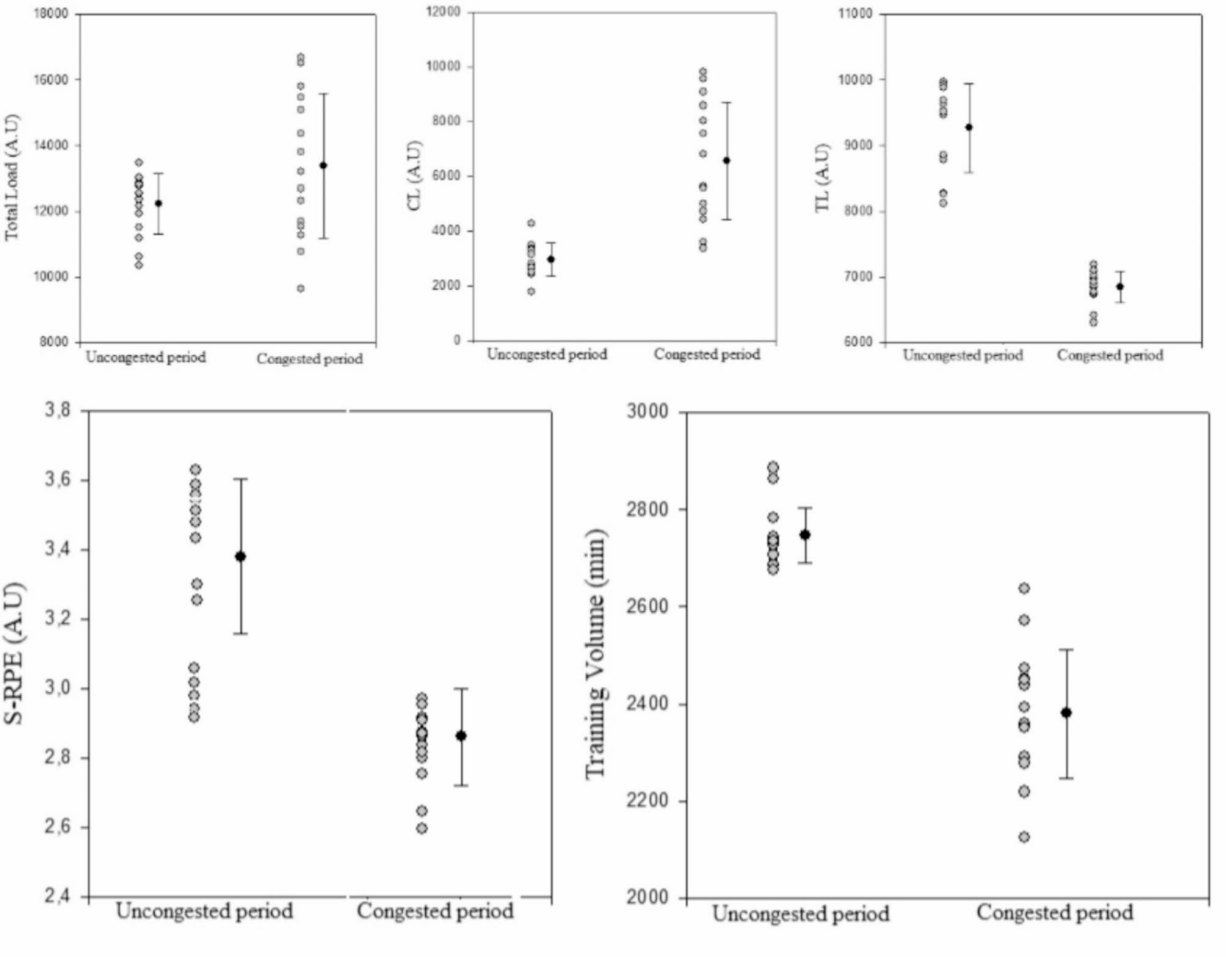
Team and individual values of workload parameters during the uncongested and congested periods. **Abbreviations: A.U**, Arbitrary units, **S-RPE**: session rating of perceived excertion. **TL: training load**,** CL**, Competitive load. Black dots and lines represent team mean values ± 95% confidence interval of different workload parameters during the uncongested and congested periods. Grey dots represent changes in individual values for different workload parameters during the uncongested and congested periods.

**Fig. 4:**
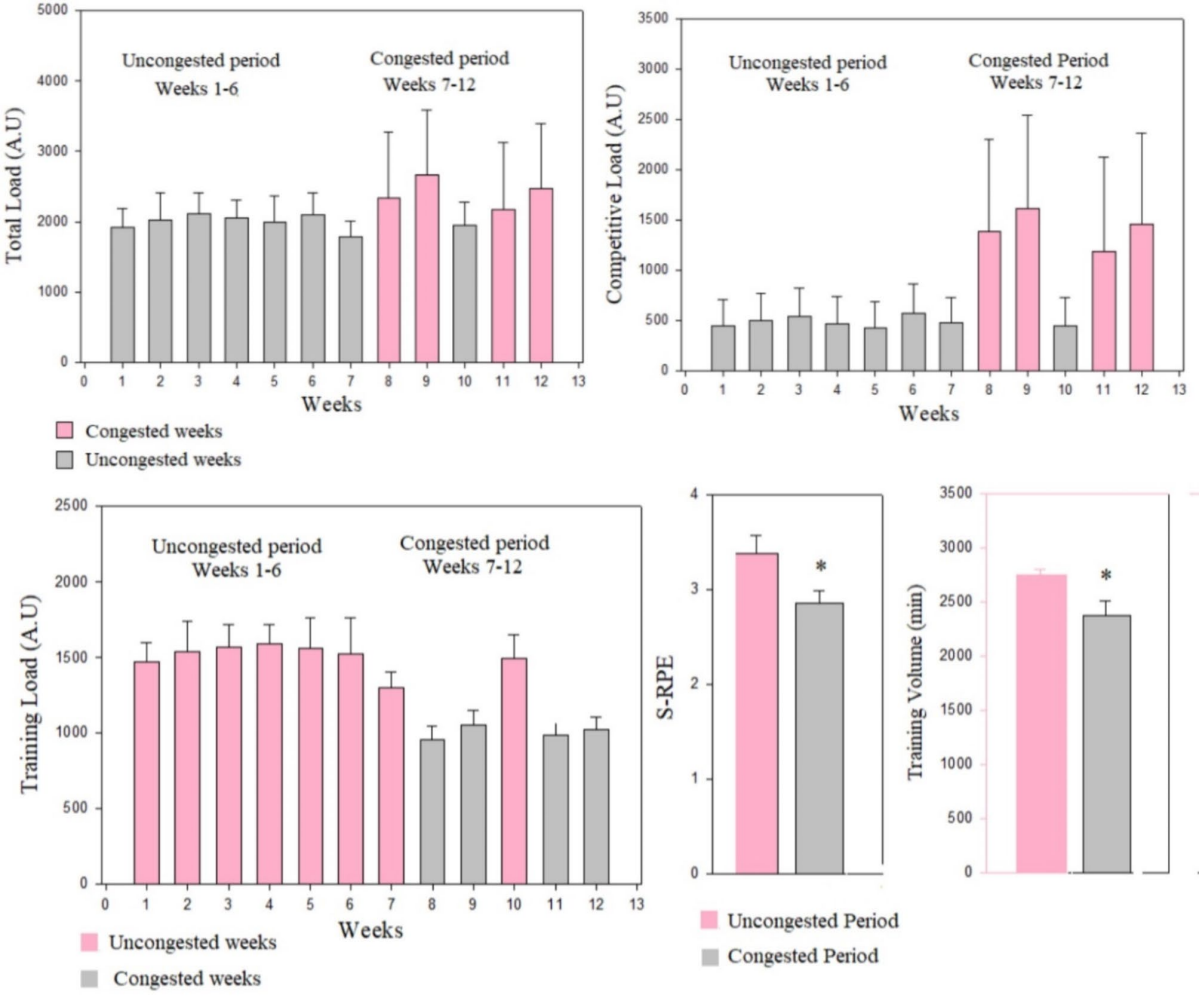
Weekly total load, competitive load, training load and S-RPE and training volume during the uncongested and congested periods. **Abbreviations: A.U**, arbitrary units, **S-RPE**: session rating of perceived exertion. **Training Load (A.U) = S-RPE (A.U) × training volume (min)**.

Workload data during the uncongested and congested periods are presented in Table 3 and illustrated in Figures 2 and 3, and 4. As a team, there were large and significant increases in total and competitive loads from the uncongested period to the congested period (ES= -0.81 to-1.84). Moreover, we observed significant and very large decreases in training load, training volume and S-RPE from the uncongested period to the congested period (ES = 2.4 to3.15).

**Table 3:** Workload during the 12-weeks training period seperated in uncongested and congested periods of match play.

Training periods	Uncongested period from T1 to T2 (*n* = 24), missing (*n* = 9; 37,5%)	Congested period from T2 to T3 (*n* = 24), missing (*n* = 9; 37,5%)	*p*-value	ES (Cohen’s d) ES_1 − 2_ –ES_2 − 3_
Total load (A.U)	12213.76 ± 924.30	13680.30 ± 1951.43	0.007*	-0.81
TL (A.U)	9271.10 ± 679.93	6827.63 ± 197.57	˂ 0.001*	3.15
CL (A.U)	2954.66 ± 604.11	6580.66 ± 2147.19	˂ 0.001*	-1.84
Training volume (min)	2746.89 ± 57.35	2380.64 ± 132.99	˂ 0.001*	2.39
S-RPE (A.U)	3.38 ± 0.22	2.86 ± 0.14	˂ 0.001*	1.74

Data are means and standard deviations. **A.U**, arbitrary units; **CL**, competitive load; **ES**, effect size; **TL**, training load. **S-RPE**, session-rating of perceived exertion (A.U).

*significant difference between uncongested and congested periods, *p* < 0.05.

The weekly total and competitive loads were significantly higher during congested weeks compared to the uncongested weeks. The weekly training load parameters were significantly higher in the uncongested weeks compared with congested weeks, and the S-RPE and training volumes were significantly greater in the uncongested period compared with the congested period (Fig. 4).

Individually, all players appear to present a higer value of CL in the congested period compared to uncongested period. The coefficient of variation of CL ranged between 23.7 and 92.9%. In addition, we showed for most of the soccer players (*n* = 13) a significant increase of total load from the uncongested period to congested periods.The coefficient of variation of total load ranged between 2.4 and 21.2%. In addition, all players present lower values of training load, S-RPE and training volume in the congested period compared to the uncongested period. The coefficients of variations ranged between 10.2 and 29.2% for the training load, between 2.4 and 17.7% for the training volume, and between 2.5 and 18.8% for S-RPE.

Mixed linear model analysis indicated a significant change in physical fitness measures (Tables 4a-b). There were significant and very large decreases in VO_2max_ and YYRL1 performances from T2 to T3 (ES = 2.73) and a significant moderate increase from T1 to T2 (ES= -0.71). In addition, we observed a significant moderate decrease in 20-meter sprint time from T2 and T3 (ES= -0.76) and a significant moderate increase from T1 to T2 (ES = 0.62). We observed a small and significant increase in SJ performance from T2 to T3 (ES = 0.21). In addition, there were no significant changes in CMJ heights between the three test sessions, and the SJ height did not significantly change from T1 to T2.

**Table 4:** Changes in physical fitness.

Competitive period	T1	T2 (*n* = 24), missing (*n* = 9 ; 37.5% )	T3 (*n* = 24), missing (*n* = 9 ; 37.5% )	*p*-value	ES (Cohen’s d) ES_1 − 2_–ES_2 − 3_
VO_2max_ (ml/kg/min)	55.51 ± 3.12	57.51 ± 2.80	49.86 ± 2.40	˂0.001*^,#^	-0.71 ; 2.73
YYIR1 (m)	2276.52 ± 372.34	2513.33 ± 333.78	1602.66 ± 286.24	˂0.001*^, **#**^	-0.71 ; 2.73
20-m sprint (s)	3.84 ± 0.14	3.73 ± 0.20	3.87 ± 0.19	0.03*^,**#**^	0.62 ; -0.76
SJ (cm)	36.81 ± 3.94	36.62 ± 3.94	35.71 ± 4.03	0.049*	0.09 ; 0.21
CMJ (cm)	38.98 ± 98.00	39.50 ± 2.52	39.14 ± 2.38	0.65	-0.23 ; 0.11

**CMJ**, countermovement jump; **SJ**, squat jump; VO_2max_, maximal oxygen consumption; YYIR1, Yo-Yo Intermittent Recovery Test Level 1.

*Significant difference between T3 and T2 as well as T3 and T1, *p* < 0.05.

^#^Significant difference between T2 and T1, *p* < 0.05.

**Table 4a:** Results of the mixed linear model for physical fitness measures.

Competitive period	T1(*n* = 24), missing (*n* = 9 ; 37.5% )	T2(*n* = 24), missing (*n* = 9 ; 37.5% )	T3(*n* = 24), missing (*n* = 9 ; 37.5% )	F	*p*-value
VO_2max_ (ml/kg/min)	55.51 ± 3.12	57.51 ± 2.80	49.86 ± 2.40	55.72	˂0.001*^,#^
YYIR1 (m)	2276.52 ± 372.34	2513.33 ± 333.78	1602.66 ± 286.24	55.72	˂0.001*^, **#**^
20-m sprint (s)	3.84 ± 0.14	3.73 ± 0.20	3.87 ± 0.19	3.93	0.02*^,**#**^
SJ (cm)	36.81 ± 3.94	36.62 ± 3.94	35.71 ± 4.03	3.35	0.04*
CMJ (cm)	38.98 ± 98.00	39.50 ± 2.52	39.14 ± 2.38	0.74	0.45

**Table 4b:** Estimates of fixed effects for the mixed linear model for physical fitness measures.

**Parameters**	**Beta coefficient**	Standard Error	**T**	p	95%CI	
					Lower	Upper
VO_2max_						
Intercept T1 T2	54.29 1.22 3.21	0.57 0.43 0.43	94.21 2.81 7.40	˂ 0.001 0.009 ˂ 0.001	54.10 56.09 48.44	56.93 58.92 51.27
**YYIRL1**						
Intercept T1 T2	2130.66 145.33 328.66	68.61 51.67 51.67	31.05 2.81 7.40	˂ 0.001 0.009 ˂ 0.001	2107.65 2344.98 1434.32	2444.34 2681.67 1771.01
**20-m sprint**						
Intercept T1 T2	3.82 0.03 -0.08	0.037 0.031 0.031	104.21 0.95 -2.76	˂ 0.001 0.34 0.01	3.75 3.63 3.78	3.94 3.82 3.97
**SJ**						
Intercept T1 T2	35.96 0.53 0.17	0.99 0.28 0.28	36.00 1.87 0.60	˂ 0.001 0.07 0.55	34.45 34.09 33.22	38.52 38.16 37.29
**CMJ**						
Intercept T1 T2	38.95 -0.29 0.28	0.58 0.27 0.27	66.16 -1.07 1.04	˂0.001 0.29 0.30	37.37 37.96 37.68	39.92 40.51 40.23

**Fig. 5:**
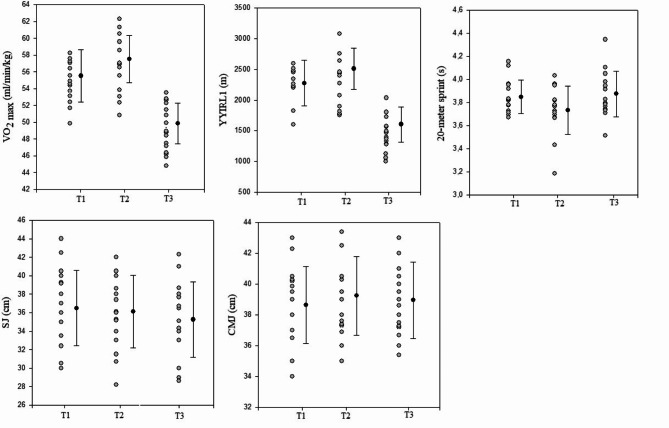
Team and individual values of physical fitness measures during the competitive periods. **Abbreviation CMJ**, countermovement jump; **SJ**, squat jump; VO_2max_, maximal oxygen consumption; YYIR1, Yo-Yo Intermittent Recovery Test Level 1.** T1**, before the uncongested period; **T2**, after the uncongested period (before the congested period);** T3**, after the congested period. Black dots and lines represent team mean values ± 95% confidence interval for physical fitness measures during the competitive periods. Grey dots represent changes in individual values for physical fitness during the competitive periods.

Individually, we showed for most of the soccer players a significant decrease in VO_2max_ and YYIRL1 values from T2 to T3 (*n* = 13) and a significant increase from T1 to T2 (*n* = 9). The coefficient of variation (CV) ranged between 5.7 and 10.3% for the VO_2 max_ and between 16.7 and 34% for the YYIRL1 performance. Most players presented lower values for 20-meter linear sprint (*n* = 9) and SJ performances (*n* = 10) in T3 compared to T1 and T2. The CV ranged between 1.4 and 8.5% for the 20 m-sprint test and between 1.2 and 8.3% for the SJ test.(Fig. 5).

**Fig. 6:**
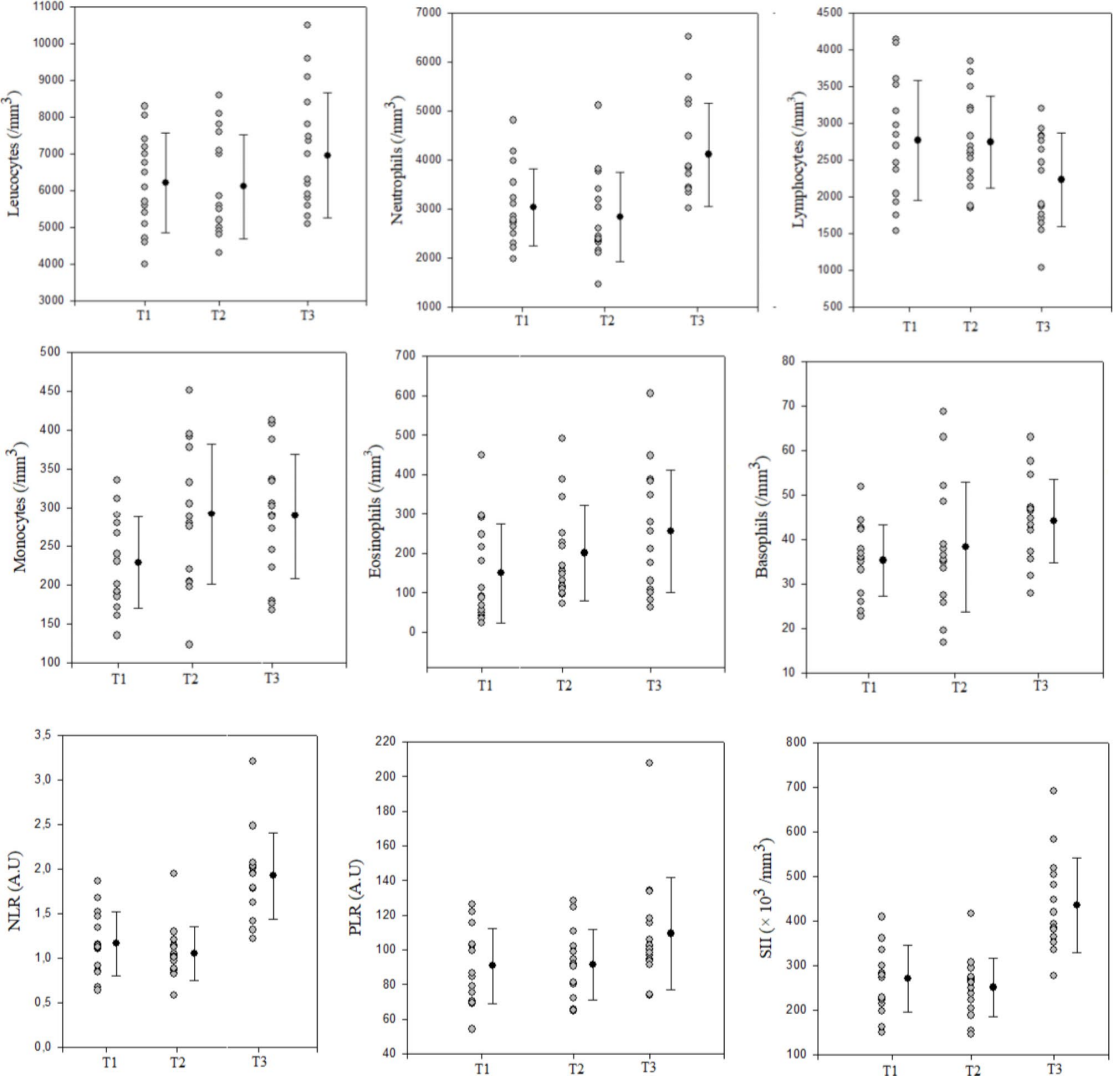
Team and individual values of cellular immune inflammation markers during the competitive periods. **Abbreviations: A.U**, Arbitrary units, **NLR**, neutrophil-to-lymphocyte ratio; **PLR**, platelet-to-lymphocyte ratio; **SII**, systemic immune inflammation index. **T1**, before the uncongested period; **T2**, after the uncongested period (before the congested period); **T3**, after the congested period. Black dots and lines represent team mean values ± 95% confidence interval of different immune inflammation markers during the competitive periods. Grey dots represent changes in individual values for different immune inflammation markers.

As a group, markers of cellular immune inflammation significantly changed during the competitive periods (Tables 5a‒b). There were significant and moderate increases in leucocytes (ES= -0.49 to -0.56), neutrophils (ES= -0.64 to -0.43), and eosinophils (ES=-0.78 to -0.40) from T1 to T3. Moreover, we observed a significant and small increase in lymphocyte counts from T1 to T3 (ES= -0.25 to -0.48). Values for N/L, PLR, and SII were significantly greater at T3 compared to T2 and T1 (Fig.6). However, counts of leucocytes, neutrophils, lymphocytes, NLR, PLR, and SII did not significantly differ between T1 and T2. In addition, we observed a significant and moderate increase in monocyte counts from T1 to T3 (ES= -0.77).

**Table 5:** Changes in cellular immune inflammation markers.

Competitive periods / cellular immune markers	T1	T2	T3	*p*-value	ES (Cohen’s d) ES_1 − 3_–ES_2 − 3_
Leucocytes (/mm^3^)	6220.70 ± 1350.76	6113.33 **±** 1419.69	6690.00 ± 1707.88	0.007*^,#^	-0.49 ; -0.56
Neutrophils (/mm^3^)	3038.47 ± 789.4	2838.21 ± 906.80	4115.16 ± 1055.11	0.04*^,#^	-0.64 ; -0.43
Lymphocytes (/mm^3^)	2763.05 ± 710.17	2752.37 ± 650.98	2215.72 ± 655.04	0.038*^,#^	-0.25 ; -0.48
Monocytes (/mm^3^)	228.71 ± 58.95	292.07 ± 90.72	313.13 ± 120.22	0.003^#^†	-0.77 ; 0.04
Eosinophils (/mm^3^)	149.20 ± 125.55	200.42 ± 121.50	254.88 ± 155.55	0.002*†	-0.78 ; -0.40
Basophils (/mm^3^)	35.31 ± 8.02	38.30 ± 14.57	44.16 ± 9.31	0.074	-0.80 ; -0.53
Platelets (× 10^3^/mm^3^)	237.80 ± 42.31	243.40 ± 43.52	230.60 ± 46.01	0.32	0.16 ; 0.29
NLR (A.U.)	1.16 ± 0.35	1.04 ± 0.30	1.92 ± 0.48	˂ 0.001*^,#^	-0.99 ; -1.01
PLR (A.U.)	90.70 ± 21.52	91.52 ± 20.35	109.35 ± 32.42	0.015*^,#^	-0.21 ; -0.44
SII (× 10^3^/mm^3^)	270.68 ± 75.62	250.95 ± 66.40	434.77 ± 106.59	˂ 0.001*,^#^	-0.75 ; -0.71

**AU**, arbitrary units; **ES**, effect size; **NLR**, neutrophil-to-lymphocyte ratio; **PLR**, platelet-to-lymphocyte ratio; **SII**, systemic immune inflammation index.

* significant differences between T2 and T3, *p* < 0.05.

^#^ significant difference between T3 and T1, *p* < 0.05.

† significant differences between T1 and T2, *p* < 0.05.

**Table 5a:** Results of the mixed linear model for the cellular immune inflammation markers.

Competitive periods / cellular immune markers	T1 (*n* = 24), missing (*n* = 9 ; 37.5% )	T2 (*n* = 24), missing (*n* = 9 ; 37.5% )	T3 (*n* = 24), missing (*n* = 9 ; 37.5% )	F	*p*-value
Leucocytes (/mm^3^)	6220.70 ± 1350.76	6113.33 **±** 1419.69	6690.00 ± 1707.88	5.92	0.007*^,#^
Neutrophils (/mm^3^)	3038.47 ± 789.4	2831.21 ± 906.80	4115.16 ± 1055.11	18.63	˂0.001*^,#^
Lymphocytes (/mm^3^)	2763.05 ± 710.17	2752.37 ± 650.98	2215.72 ± 655.04	7.38	0.003*^,#^
Monocytes(/mm^3^)	228.71 ± 58.95	292.07 ± 90.72	313.13 ± 120.22	7.32	0.003*†
Eosinophils (/mm^3^)	149.20 ± 125.55	200.42 ± 121.50	254.88 ± 155.55	5.25	0.01*†
Basophils (/mm^3^)	35.31 ± 8.02	38.30 ± 14.57	44.16 ± 9.31	5.21	0.01
Platelets (× 10^3^/mm^3^)	237.80 ± 42.31	243.40 ± 43.52	230.60 ± 46.01	1.18	0.32
NLR (A.U.)	1.16 ± 0.35	1.04 ± 0.30	1.92 ± 0.48	6.33	0.005
PLR (A.U.)	90.70 ± 21.52	91.52 ± 20.35	109.35 ± 32.42	4.01	0.029*^,#^
SII (× 10^3^/mm^3^)	270.68 ± 75.62	250.95 ± 66.40	434.77 ± 106.59	22.18	˂ 0.001*,^#^

**AU**, arbitrary units; **ES**, effect size; **NLR**, neutrophil-to-lymphocyte ratio; **PLR**, platelet-to-lymphocyte ratio; **SII**, systemic immune inflammation index.

* significant differences between T2 and T3, *p* < 0.05.

^#^ significant difference between T3 and T1, *p* < 0.05.

† significant differences between T1 and T2, *p* < 0.05.

**Table 5b:** Estimates of fixed effects for the mixed linear model for the cellular immune inflammation markers.

Parameters	Beta coefficient	Standard Error	T	p	95% CI	
					Lower	Upper
Leucocytes						
Intercept T1 T2	6431.11 -211.11 -317.77	355.28 154.68 154.68	18.10 -1.36 -2.05	˂ 0.001 0.18 0.04	5460.51 5353.85 6200.51	6979.48 6872.81 7719.48
**Neutrophils**						
Intercept T1 T2	3330.61 -292.14 -492.40	199.98 129.88 129.88	16.65 -2.24 -3.79	˂ 0.001 0.033 ˂ 0.001	2571.09 2370.83 3647.78	3505.85 2305.59 4582.54
**Lymphocytes**						
Intercept T1 T2	2581.75 186.54 162.56	155.86 90.92 90.92	16.56 2.05 1.78	˂ 0.001 0.05 0.08	2414.62 2390.64 1878.97	3.121.96 3097.99 2586.32
**Monocytes**						
Intercept T1 T2	269.88 -41.17 22.18	16.95 10.77 10.77	15.92 -3.82 2.06	˂ 0.001 ˂ 0.001 0.049	189.34 252.70 249.50	268.07 331.43 328.31
**Eosinophils**						
Intercept T1 T2	201.50 -52.29 -1.08	29.35 18.82 18.82	6.86 -2.77 -0.05	˂0.001 0.01 0.95	80.85 132.07 186.53	217.55 268.76 323.23
**Basophils**						
Intercept T1 T2	39.26 -3.94 -0.95	2.34 1.61 1.61	16.76 -2.45 -0.59	˂0.001 0.021 0.55	29.74 32.73 38.59	40.88 43.87 49.73
**Platelets**						
Intercept T1 T2	39.26 -3.94 -0.95	2.34 1.61 1.61	16.76 -2.45 -0.59	˂0.001 0.021 0.55	29.74 32.73 38.59	40.88 43.87 49.73
**NLR**						
Intercept T1 T2	1.37 -0.21 -0.33	0.05 0.08 0.08	23.79 -2.63 -4.02	˂0.001 0.01 ˂0.001	0.96 0.85 1.72	1.36 1.24 2.12
**PLR**						
Intercept T1 T2	97.19 -6.49 -5.66	4.94 4.29 4.29	19.67 -1.51 -1.31	˂0.001 0.14 0.19	77.87 78.69 96.52	103.53 104.35 122.18
**SII**						
Intercept T1 T2	318.80 -48.12 -67.84	13.09 17.49 17.49	24.34 -2.75 -3.87	˂0.001 0.01 ˂0.001	77.87 78.69 96.52	103.53 104.35 122.18

Abbreviations : NLR, neutrophil-to-lymphocyte ratio; PLR, platelet-to-lymphocyte ratio; SII, systemic immune inflammation index

Individually, most of the players presented higher values of leucocytes (*n* = 13), neutrophils (*n* = 14), eosinophils (*n* = 13), basophils (*n* = 13), and monocytes (*n* = 9) at T3 as compared to T1 and T2. The coefficient of variation ranged between 4.6 and 18.4% for leucocyte counts, between 14.5 and 39.4% for neutrophil counts, between 8.5 and 53.9% for the monocyte counts, between 9.6 and 46.5% for the eosinophil counts and between 7.7 and 29.8% for the basophil counts. In addition, most players presented higher values of NLR (*n* = 14), PLR (*n* = 9) and SII (*n* = 14) at T3 as compared to T1 and T2. The coefficient of variation ranged between 23.8 and 91.6% for NLR, between 11.8 and 57.6% for PLR and between 17.4 and 93.3% for SII values (Fig. 6).

Linear regression analyses indicated no significant relations between markers of cellular immune inflammation and work load parameters after the non-congested period (Table 6a). Of note, total load accounted for the significant changes in the variance of leucocyte counts (*r* = 0.53, R²= 0.28, *p* = 0.04) during the congested period. In addition, training load accounted for changes in NLR values (*r* = 0.50; R²= 0.27, *p* = 0.049) during the congested period (Table 6a).

**Table 6a:** Associations between workload parameters and markers of cellular immune inflammation in elite soccer players.

Uncongested period (T1-T2)					Congested period (T2-T3)						
		Total load (AU)	Training load (AU)	CL (AU)	S-RPE (AU)	TR-V (min)	Total load (AU)	Training load (AU)	CL (A.U)	S-RPE	TR-V
Leuc (/mm^3^)	r R² p	0.17 0.02 0.54	0.32 0.10 0.24	0.14 0.01 0.60	0.45 0.20 0.09	0.38 0.14 0.15	0.53 0.28 0.04*	-0.16 0.02 0.55	0.22 0.04 0.42	0.20 0.04 0.45	0.12 0.01 0.66
NLR (A.U.)	r R² p	0.17 0.02 0.53	0.22 0.04 0.43	0.04 0.001 0.86	0.12 0.01 0.66	0.19 0.03 0.48	-0.21 0.04 0.44	0.50 0.25 0.049*	0.06 0.003 0.80	0.26 0.07 0.33	0.29 0.08 0.28
PLR (A.U.)	r R² p	-0.36 0.129 0.18	-0.26 0.06 0.34	0.09 0.008 0.72	0.23 0.05 0.39	0.19 0.03 0.49	-0.07 0.004 0.78	0.16 0.02 0.56	-0.01 0.0001 0.97	0.05 0.002 0.85	0.06 0.003 0.83
SII (× 10^3^ /mm^3^)	r R² p	0.19 0.03 0.49	-0.18 0.03 0.52	0.18 0.03 0.51	0.2 0.05 0.39	0.130.01 0.63	0.01 0.0001 0.95	0.16 0.02 0.55	0.20 0.04 0.47	0.12 0.01 0.65	0.27 0.07 0.32

**AU**, arbitrary units; **CL**, competitive load; **NLR**, neutrophil-to-lymphocyte ratio; **PLR**, platelet-to-lymphocyte ratio; **SII**, systemic immune inflammation index; **TL**, training load; **S-RPE**, session-rating of perceived exertion (A.U). **TR-V**, training volume. *significant correlation between parameters, *p* < 0.05.

**Table 6b:** Within-subject correlation between cellular immune inflammation markers and workload parameters during the competitive periods.

Paremeters	Leucocytes			NLR			PLR			SII		
	*P*	*r* (95%CIs)	Description	*P*	*r* (95%CIs)	Description	*P*	*r* (95%CIs)	Description	*P*	*r* (95%CIs)	Description
Total load	0.49	-0.13 (-0.46 0.24)	Unclear	0.22	-0.22 (-0.54 ; 0.14)	Unclear	0.77	-0.05 (-0.40 ; 0.31)	Unclear	0.24	-0.28 (-0.61; 0.04)	Unclear
Competitive load	0.01	0.53 (0.07; 0.64)	Clear	˂0.001	0.59 (0.30; -0.78)	Clear	0.18	0.25 (-0.12 ; 0.56)	Unclear	˂0.001	0.64 (0.36 ; 0.81)	Clear
Training load	0.43	0.27 (-0.54 ; 0.90)	Unclear	˂0.001	-0.65 (-0.81 ; -0.37)	Clear	0.07	-0.32 (0.61 ; 0.03)	Unclear	˂0.001	-0.68 (-0.82; -0.38)	Clear
S-RPE	0.41	-013 (-0.47 ; 0.23)	Unclear	0.001	-0.67 (-0.78: -0.28)	Clear	0.09	-0.31 (-0. 60 ; 0.05)	Unclear	0.001	-0.58 (− 0.78 − 0.28)	Clear
Training volume	0.21	-0.23 (-0.54 ; 0.13)	Unclear	0.001	-0.60 (-0.30 ; -0.78)	Clear	0.13	-028 (-0.58 ; 0.08)	Unclear	0.001	0.61 (-0.79 ; -0.32)	Clear

**AU**, arbitrary units; **CL**, competitive load; **NLR**, neutrophil-to-lymphocyte ratio; **PLR**, platelet-to-lymphocyteratio; **SII**, systemic immune inflammation index; **TL**, training load; **S-RPE**, session-rating of perceived exertion (A.U). **TR-V**, training volume.

In the within-subjets correlational analysis, we showed a moderate correlation between leucocyte count and CL (*r* = 0.53; *p* = 0.01) during the competitive periods. In addition, a large clear correlation was observed between NLR and SII with CL (r= [0.59–0.64; p˂ 0.001), TL (r= [0.65–0.68]; p˂ 0.001), S-RPE (r= [0.65–0.68]; *p* = 0.001), and training volume (r= [0.60–0.61; *p* = 0.001). (Table 6b).

**Fig. 7:**
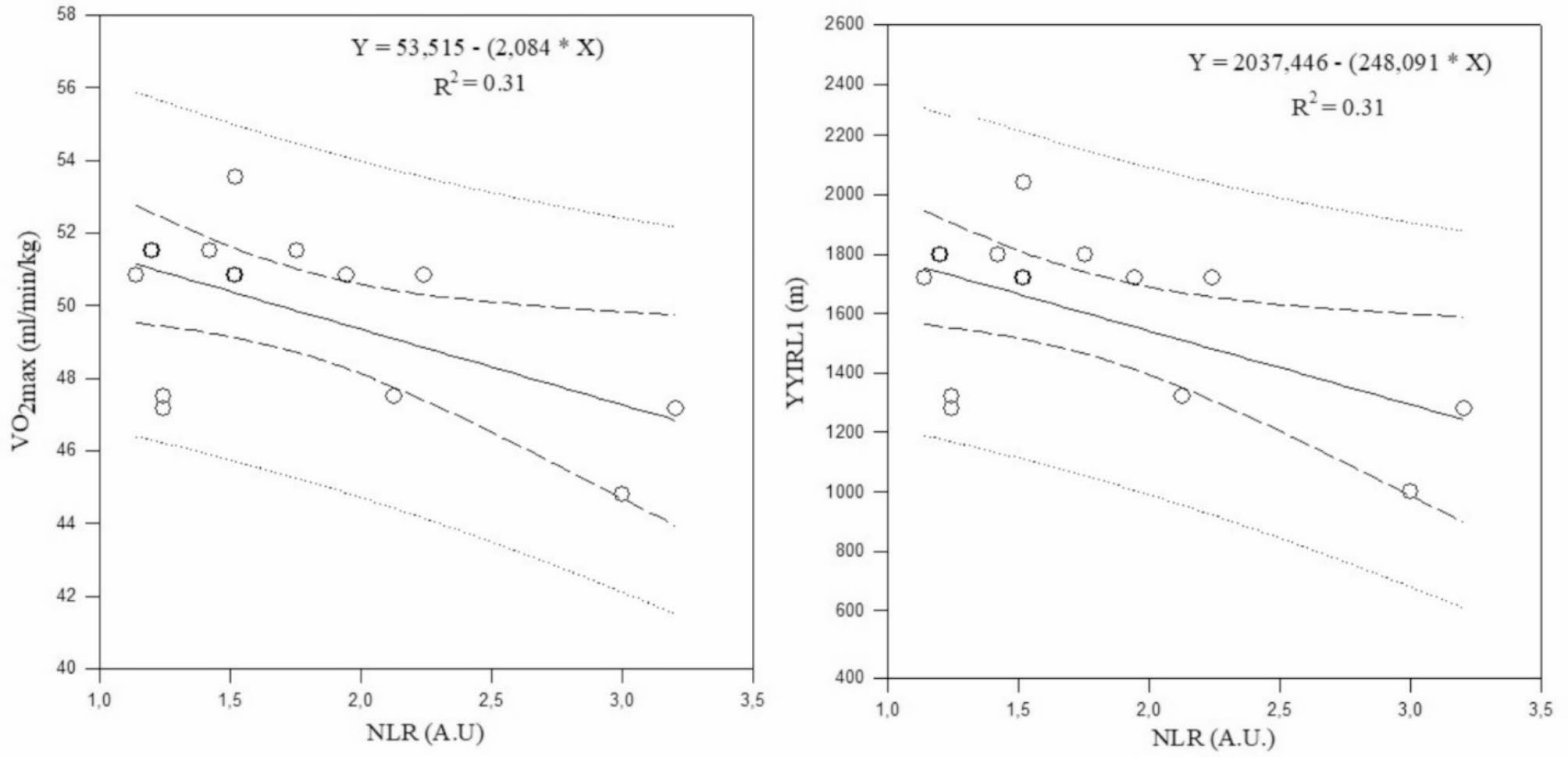
Associations between NLR with VO_2max_ and YYIRL1 performance during the congested period. **Abbreviations: NLR**, neutrophil-to-lymphocyte ratio; **VO**_**2max**,_ maximal oxygen consumption; **YYIR1**, Yo-Yo Intermittent Recovery Test Level 1.

**Fig. 8:**
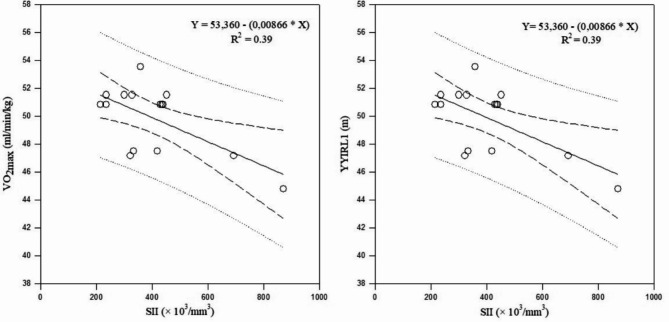
Associations between SSI with VO_2max_ and YYIRL1 performance during the congested period. **Abbreviations: SII**, systemic immune inflammation index; **VO**_**2max**,_ maximal oxygen consumption; **YYIR1**, Yo-Yo Intermittent Recovery Test Level 1.

The observed associations between markers of cellular immune inflammation and physical fitness are summarized in Table 7a. There were no significant correlations between these markers and physical fitness after the uncongested period. Values of YYIRL1 performance were negatively correlated with leucocyte counts (*r*= -0.63; *p* = 0.01), NLR (*r*= -0.56; *p* = 0.02) and SII (*r*= -0.63; *p* = 0.01) after the congested period, while VO_2max_ was negatively correlated with leucocyte counts (*r*= -0.63; *p* = 0.01), NLR (*r*= -0.56; *p* = 0.02), and SII (*r*= -0.63; *p* = 0.01). Linear regression analysis suggested that NLR accounted for the significant changes of the variances in YYIRL1 and VO_2max_ after the congested period (R²= 0.31; *p* = 0.03; Fig. 7). In addition, SII accounted for the significant changes in the variance in YYIRL1 and VO_2max_ after the congested period (R²= 0.39; *p* = 0.01; Fig. 8).

**Table 7a:** Associations between cellular immune inflammation markers and physical fitness.

		Uncongested period (T1-T2)					Congested period (T2-T3)				
		YYIRL1 (m)	VO_2max_ (ml/min/kg)	20-m sprint (s)	SJ (cm)	CMJ (cm)	YYIRL1 (m)	VO_2max_ (ml/min/kg)	20-m sprint (s)	SJ (cm)	CMJ (cm)
Leucocytes (/mm^3^)	r R² p	0.2 0.04 0.82	0.2 0.04 0.82	0.01 0.001 0.95	0.02 0.004 0.91	0.04 0.008 0.74	-0.63 0.39 0.01*	-0.63 0.39 0.01*	0.4 0.16 0.70	0.16 0.02 0.56	0.34 0.11 0.20
NLR (A.U)	r R² p	0.12 0.01 0.65	0.19 0.03 0.49	0.03 0.009 0.90	0.41 0.16 0.12	0.41 0.16 0.09	-0.56 0.31 0.03*	-0.56 0.31 0.03*	0.19 0.03 0.47	0.06 0.03 0.82	0.20 0.04 0,46
PLR (A.U)	r R² p	0.48 0.23 0.06	0.48 0.23 0.06	0.1 0.01 0.96	0.28 0.07 0.30	0.09 0.008 0.72	-0.39 0.15 0.14	-0.39 0.15 0.14	0.11 0.01 0.67	0.26 0.06 0.34	0.3 0.09 0.74
SII (× 10^3^/mm^3^)	r R² p	0.38 0.14 0.16	0.38 0.14 0.16	0.1 0.001 0.96	0.42 0.17 0.11	0.42 0.17 0.09	-0.63 0.39 0.01*	-0.63 0.39 0.01*	0.06 0.003 0.90	0.16 0.02 0.56	0.34 0.11 0.21

**AU**, arbitrary units; ,** NLR**, neutrophil-to-lymphocyte ratio, **PLR**, platelet-to-lymphocyte ratio, **SII**, systemic immune inflammation index; **TL**, training load; **CL**, competitive load; **CMJ**, countermovement jump; **SJ**, squat jump; **YYIR1**, Yo-Yo intermittent recovery test level 1.**VO**_**2max**_, maximal oxygen consumption. *significant correlation between parameters, *p* < 0.05.

**Table 7b:** Within-subject correlation between cellular immune inflammation markers and workload parameters.

Paremeters	Leucocytes (AU)			NLR (AU)			PLR (AU)			SII (A.U)		
	*P*	*r* (95%CIs)	Description	*P*	*r* (95%CIs)	Description	*P*	*r* (95%CIs)	Description	*P*	*r* (95%CIs)	Description
VO_2 max_	0.007	-0.25 (-0.16 ; -0.52)	Clear	˂ 0.001	-0.69 (-0.30 ; -0. 78)	Clear	0.03	-0.38 (-0.65; -0.30)	Clear	˂ 0.001	-0.66 (-0.82 ;-0.41)	Clear
YYIRL1	0.007	-0.25 (-0.23; -0.52)	Unclear	˂ 0.001	-0.69 (-0.30 ; -0. 78)	Clear	0.03	-0.38 (-0.65; -0.30)	Clear	˂ 0.001	-0.66 (-0.82 ;-0.41)	Clear
20-meter sprint	0.49	0.10 (-0.19 ; 0.38)	Unclear	0.45	0.18 (-0.27 ; 0.44)	Unclear	0.90	0.01 (-0.27 ; 0.31)	Unclear	0.29	0.13 (-0.16 ; 0.41)	Unclear
SJ	0.73	-0.05 (-0.34 ; 0.24)	Unclear	0.67	-0.24 (-0.30 ; 0.41)	Unclear	0.59	-0.082 (-0.36 ; 0.21)	Unclear	0.41	-0.17 (-0.44 ; 0.13)	Unclear
CMJ	0.21	0.18 (-0.11 ; 0.45)	Unclear	0.70	-0.07 (-0.42 ; 0.29)	Unclear	0.17	-0.20 (-0.47 ; 0.09)	Unclear	0.80	-0.03 (-0.32 ; 0.25)	Unclear

**AU**, arbitrary units; **NLR**, neutrophil-to-lymphocyte ratio, **PLR**, platelet-to-lymphocyte ratio, **SII**, systemic immune inflammation index; **TL**, training load; **CL**, competitive load; **CMJ**, countermovement jump; **SJ**, squat jump; **YYIR1**, Yo-Yo intermittent recovery test level 1.**VO**_**2max**_, maximal oxygen consumption.

The within-subject correlation between cellular immune inflammation markers and physical fitness performance are presented in Table 7b. We observed a small correlation between leucocyte count with VO2max and YYIRL1 performance (*r*= -0.25;*p* = 0.007). The NLR and SII were moderatly correlated with VO_2max_ and YYIRL1 performance (*r*= -0.69; p ˂ 0.001; *r*= -0.66, p ˂ 0.001). In addition, we showed a small correlation between PLR with VO_2max_VO_2max_and YYIRL1 performance (*r*= -0.38; *p* = 0.03).

## Discussion

This study investigated fluctuations in the markers of inflammation as related to the physical fitness of elite soccer players after training and match exposure during the competitive periods. The main finding was that most elite players presented an increase in leucocyte counts and its subsets (neutrophil, eosinophil, monocyte, Basophil) and markers of immune inflammation (NLR, PLR, and SII) after the congested period of match play. We were also able to show decreases in lymphocyte counts and physical fitness performance levels after a congested period. Furthermore, markers of immune inflammation responses (NLR, PLR and SII) were negatively correlated with aerobic fitness performance (YYIRL1, VO_2max_).

Conversely, resting values of leucocytes, neutrophils, and lymphocyte counts and markers of immune inflammation were unchanged after the uncongested playing period of six weeks. The absence of a significant change in these markers in conjunction with improvements in physical performance (YYIR1, VO_2max_, 20-m sprint) during this period indicates a positive and thus performance enhancing training strategy. In contrast, congested periods of matches affected the players’ immune system as indicated by increased occurrence of neutrophilia and lymphocytopenia.

As such, our study suggests that an increase in the competitive load can lead to leucocytosis and alterations of leucocyte subsets after a congested competitive schedule. Nevertheless, the decrease in performance combined with altered markers of inflammation after the congested playing period could also be explained by the chronic effects of the 12-week competitive period as well as the acute effect of the previous congested week of match play and/or the last match played during this period. Thus, potential differences in scheduling training programs and the number of high-intensity matches should not be disregarded so as to limit the effects on inflammation and leucocytes^35^. This is supported by studies reporting impaired immune cell counts in soccer players during intense match programs either due to competitive matches^36^ or vigorous training periods^37^. In fact, neutrophils and monocytes reduce infections and have important roles in the immune system. The increases in neutrophils and monocytes may result from the mobilization of marginal immune cell pools in the liver, spleen, lung, and on vessel walls mediated by hormones such as glucocorticoids and catecholamines^38,39^. In addition to affecting neutrophil and monocyte counts, catecholamines and glucocorticoids also impact immune cell function^38^. It is likely that the increased neutrophil and monocyte counts are a compensate for decreased neutrophil and monocyte functionality during the high-intensity training periods^40^.

Lymphocytes are produced by lymphoid stem cells in the bone marrow and are important components of the adaptive immune system. Lymphocytopenia after periods of match congestion could be explained by two hypotheses: an impairment in immune function due to apoptosis of lymphocytes^41^, or migration of lymphocytes from the circulation to peripheral tissue, thereby increasing immune competence and surveillance^42^.

Additionally, our study revealed that lymphocyte counts decreased with an intensified period of competition when physical performance decreased suggesting that low lymphocyte levels could potentially be used as a marker of fatigue and may reflect the residual effects of accumulated fatigue, which could be linked to insufficient recovery. The cause of this depression in acquired immunity could be related to elevated circulating stress hormones, particularly cortisol, and alterations in the pro/anti-inflammatory cytokine balance in response to increased total load and competitive loads during periods of match congestion. This assumption needs to be confirmed in future studies though. Notably, our findings are in agreement with a study by Selmi et al.^37^and Malm et al.^43^.

The present study found increases in eosinophil and basophil counts throughout the observation period, both when performance was increased after an uncongested period, and when performance decreased after the congested period. These results could be due to the role of eosinophils and basophils in allergic reactions^44^. As such, the changes in cell count during the uncongested period may be a result of either an allergic response, or they may reflect a fatigued state due to increases in total load and competitive loads.

There is currently limited use of integrative immune cellular markers such as NLR, PLR, and SII in exercise physiology, particularly as these markers can be used to monitor exercise-induced strain and recovery processes^11^. However, to our knowledge, there are no studies examining the use of these markers in elite soccer players during the competitive period. Our data indicate that NLR, PLR, and SII levels increased after a congested match play period, when total load and competitive loads were increased as reported elsewhere^11^.

In contrast to changes in leucocyte subsets, platelet counts did not change, suggesting leucocytes (and theirsubsets)responses may be more sensitive during competitive periods and soccer training programs. Furthermore, the absence of changes in platelet counts and the decrease in lymphocytes after the congested period may account for increases in PLR. A clear correlation between leucocyte counts, NLR, SIIwith workload parameters (total load, CL, TL, training volume and S-RPE) during the competitive periods were found, suggesting that these markers can be used to monitor the intensity of training and/or competition. This finding is similar to results reported by Owen et al.^36^ In contrast, PLR was not associated with workload parameters, suggesting that it may be more useful to monitor variation in NLR and SSII . We found NLR, and SII were negatively correlated with aerobic fitness performance . Furthermore, decreases in VO_2max_ and the distance covered during YYIRL1 tests were related to higher NLR and SII values. Unlike NLR and SSI, PLR was not associated with other physical fitness measures (20-meter sprint, SJ, and CMJ), suggesting that it may be more useful to monitor variations in NLR, PLR and SII to assess aerobic fitness in elite soccer players, similarly to Zacher et al.’s findings in Olympic athletes^26^.

## Limitations and future research perspectives

Our study has some limitations: (1) the lack of a true control group did not allow the identification of the specific effects of a congested period on the examined markers; (2) immune markers were evaluated 72 h after the last match during both congested and uncongested periods of match play, which could limit our understanding of the acute and residual effects of soccer training and competition on these markers. That is, measures immediately, or after 12h, 24 h, and 48 h would have presented a better overview of the delayed effects of markers of the immunological stress responses and physical fitness; (3) the limited use of cellular immune inflammation markers (NLR, PLR, and SII) constrain the interpretation of our results. (4) our study included only elite male soccer players, of a limited number, making it challenging to generalize our findings or to extrapolate them to female athletes; (5) our analysis is based on a rather small sample size which is common in elite sports, and some of our correlation coefficients, while significant, were of a weak to moderate strength (due to our reduced statistical power). Future studies should have larger sample sizes in order to strengthen the validity of our findings. Regular testing of resting values of immune inflammation markers for elite soccer players and kinetics of different soccer exercises should be established, as reference values for soccer players. Of note, kinetics of different exercise modalities are lacking thus far. Future studies should therefore focus on assessing NLR, PLR, and SII in elite soccer players and stratify values by age, sex, training status and exercise modalities to determine baseline parameters. In this context, special consideration should be given to exercise-specific variables such as hematocrit, dietary habits, hydration, and hormonal status, since these parameters likely influence baseline levels and exercise kinetics.

## Conclusions

The present findings reveal changes in immune cell counts after a congested soccer period when the innate immune cell (i.e., neutrophils, eosinophils, basophils, monocytes) increased and adaptive immune cells (i.e., lymphocytes) decreased. In addition, there were increases in cellular immune inflammation markers (i.e., NLR, PLR, and SII) that significantly correlated with reduced levels of physical fitness after congested match play periods. Levels of NLR, and SII were related to workload, suggesting the usefulness of these markers to manage training and competitive load in elite male soccer players. We also observed associations between physical fitness and SII, PLR and NLR, suggesting that these may be promising markers for monitoring physical fitness of elite male soccer players.

### Practical application

Our study is the first that combines cellular immune inflammation markers, workload parameters, and physical fitness variables during a soccer season that includes congested and uncongested periods of match play. Our observational analysis for the competitive periods showed significant associations of changes in cardiorespiratory fitness (VO_2max_, YYIRL1) with NLR, PLR and SII values. Our findings lead us to suggest the NLR, PLR and SII are useful monitoring tools for identifying decreases in aerobic performance in players exposed to highly competitive loads during the congested period. Furthermore, NLR and SII were related to workload parameters during the competitive periods, suggesting the usefulness of these markers in providing information on the impact of training and competition on physical fitness of elite soccer players. Implementing these markers into routine assessments may enable athletes and coaches to better assess individual recovery needs. These recommendations could provide useful information for coaches and medical staff in improving workload monitoring and physical fitness development to manage player work-strain ratio and to avoid overtraining.

## Data Availability

The datasets generated during and analyzed during the current study are not publicly available due to confidential information about the participants but are available from the corresponding author on reasonable request.
